# Design of a Highly Sensitive Photonic Crystal Fiber Sensor for Sulfuric Acid Detection

**DOI:** 10.3390/mi13050670

**Published:** 2022-04-25

**Authors:** Md. Ahasan Habib, Lway Faisal Abdulrazak, Musab Magam, Laiq Jamal, Khurram Karim Qureshi

**Affiliations:** 1 Department of Electrical & Electronic Engineering, Rajshahi University of Engineering & Technology, Rajshahi 6204, Bangladesh; habib.eee.116.ah@gmail.com; 2 Department of Computer Science, Cihan University-Sulaimaniya, Sulaimaniya 46001, Iraq; lway.faisal@sulicihan.edu.krd; 3 Department of Electrical Engineering, College of Engineering, University of Sulaimani, Sulaimaniya 46001, Iraq; 4 Optical Communications and Sensors Laboratory (OCSL), Department of Electrical Engineering, King Fahd University of Petroleum and Minerals, Dhahran 31261, Saudi Arabia; g201803060@kfupm.edu.sa (M.M.); g201708290@kfupm.edu.sa (L.J.); 5 Center for Communication Systems and Sensing, King Fahd University of Petroleum and Minerals, Dhahran 31261, Saudi Arabia

**Keywords:** optical sensor, relative sensitivity, numerical aperture, single-mode propagation

## Abstract

In this research, a photonic crystal fiber (PCF)-based sulfuric acid detector is proposed and investigated to identify the exact concentration of sulfuric acid in a mixture with water. In order to calculate the sensing and propagation characteristics, a finite element method (FEM) based on COMSOL Multiphysics software is employed. The extensive simulation results verified that the proposed optical detector could achieve an ultra-high sensitivity of around 97.8% at optimum structural and operating conditions. Furthermore, the proposed sensor exhibited negligible loss with suitable numerical aperture and single-mode propagation at fixed operating conditions. In addition, the circular air holes in the core and cladding reduce fabrication complexity and can be easily produced using the current technology. Therefore, we strongly believe that the proposed detector will soon find its use in numerous industrial applications.

## 1. Introduction

Sulfuric acid has become an integral constituent in numerous industries such as drug, chemical, fertilizer manufacturing plants, etc. This chemical substance has been extensively employed in thousands of production and process industries as the primary raw material for the last few decades. A massive amount of sulfuric acid is used to manufacture phosphate fertilizers, copper leaching, inorganic pigment, petroleum refining, paper production, and industrial organic chemical production [1,2]. In the case of different industrial applications, sulfuric acid with different concentrations is required, and most of the time, the required acid is made by mixing a certain amount of water with it. That is why it is essential to determine the level of dilution of acid before employing it for any production.

In recent years, numerous optoelectronics researchers proposed various waveguides such as dielectric waveguides, parallel-plate waveguides, metallic waveguides, and Bragg fibers, to name a few, to guide electromagnetic waves from source to destination [3,4,5,6,7]. The dielectric waveguides or photonic crystal fibers (PCFs) offer superior propagation characteristics, compared with the waveguides mentioned earlier [8]. The PCFs can be classified into three major categories: solid core, porous core, and hollow core. Although all types of PCFs can be effectively employed in communication applications, only porous core and hollow-core PCFs can be employed in sensing and spectroscopic applications. Hitherto, several optical fiber-based gas and liquid sensors have been proposed to operate in the terahertz [9,10,11,12,13] or visible optical regimes [14,15]. The optical sensors that operate on terahertz signals offer higher sensitivity [16,17]. Researchers have proposed using a constant refractive index through all terahertz bands, which is impractical. On the other hand, some authors proposed optical sensors where the wavelength-dependent refractive index of the analyte was considered, which ensures better reliability [14]. In that article, the authors proposed a hollow-core PCF-based alcohol sensor that offered high relative sensitivity of 89%, with negligible confinement loss at an operating wavelength of 1.55 µm. However, in the last few years, several optical fiber-based chemical sensors were proposed based on the same principle (spectroscopy) used in Ref. [14]. Researchers recently introduced different metal-coated plasmonic sensors, ensuring excellent sensing characteristics [18,19,20,21,22]. In 2019, Podder et al. proposed a hybrid core fused silica-based chemical sensor to identify the concentration level of sulfuric acid [23]. The authors claimed that the proposed sensor could achieve maximum relative sensitivity of only 64% with negligible attenuation at optimum structural conditions. However, the proposed sensor structure is very complicated and challenging to be produced in the laboratory due to different shaped air holes in the core.

This article presents a novel technique to determine the concentration of sulfuric acid diluted in water using a PCF. A simple structured hollow-core PCF-based sensor is proposed and numerically investigated to detect the actual concentration level of the sulfuric acid solution with water. The optimum design and operating conditions are selected using a trial-and-error method. The numerical analysis ensures that the proposed sensor can provide excellent sensing characteristics under those conditions. As a result, the maximum relative sensitivity of the proposed sensor is almost 97.8%, with very negligible confinement loss on the order of 10^−7^ dB/m. In addition, the single-mode PCF also offers favorable numerical aperture, spot size, and effective core area at optimal conditions.

## 2. Geometry of the Proposed Sensor

**Figure 1:**
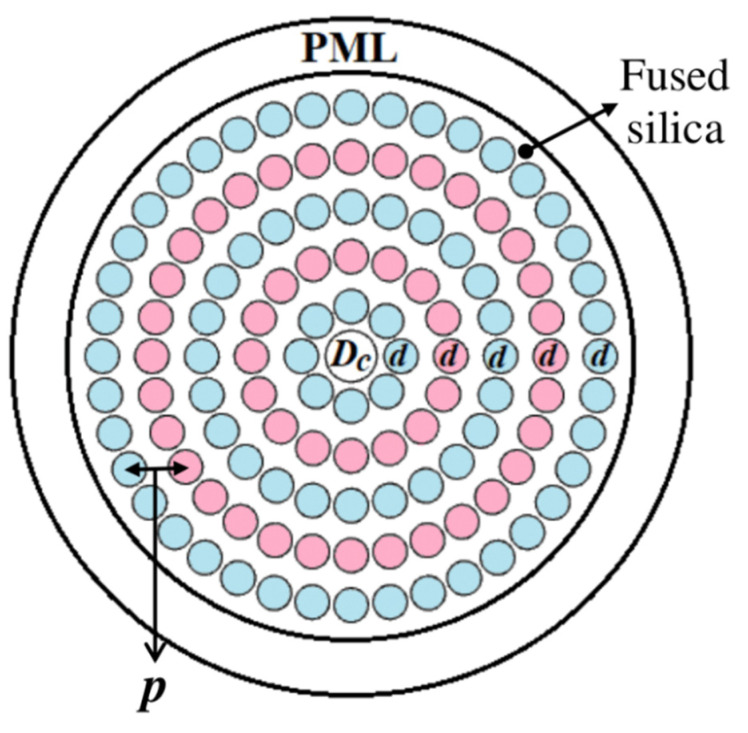
A 2D view of the proposed sulfuric acid concentration level detector.

Figure 1 illustrates the two-dimensional view of the proposed sulfuric acid concentration level detector. In the shown structure, a single channel hollow core is proposed, where the sensing analyte is injected, and the diameter of this channel is denoted by Dc. In the cladding section, to create a dielectric environment around the core, five rings of circular air holes are proposed, where the diameter of each air hole is symbolized by d. The minimum distance between two successive air hole rings is called pitch and is represented by the symbol p. The ratio between the d and p is called air filling fraction (AFF) and is maintained throughout the simulation process. To avoid the complexity of realization of the proposed sensor and for better understanding of the readers, all the parametric values are related to only parameter p, where, Dc = 1.2 p and d = 0.6 p. By using the trial and error method, the optimum value of p was found to be 3 µm. It was preserved throughout the numerical analysis process.

When the light beam travels through the core of the fiber, a fraction of light diverges from the core and propagates towards the outer surface of the waveguide. After that, a fraction of that light signal returns to the core due to this back reflection. A circular perfectly matched layer (PML) boundary condition was used to avoid this undesirable situation, whose primary function is to absorb the light incident upon it. Finally, fused silica was selected as the base material of the proposed sensor due to its supreme advantages over other materials in the optical domain. The light-dependent refractive index (RI) of the background material is calculated using the Sellmier equations as follows [14]:

(1) $$n=1+8.06051\times 10^{-5}+\frac{2.480990\times 10^{-2}}{132.274-\lambda^{-2}}+\frac{1.74557\times 10^{-4}}{39.32957-\lambda^{-2}}\;\left(\mathrm{for}\;\mathrm{air}\right)$$

(2) $$n^{2}=1+\frac{0.69617\lambda^{2}}{\lambda^{2}-0.0684^{2}}+\frac{0.40794\lambda^{2}}{\lambda^{2}-0.11624^{2}}+\frac{0.89748\lambda^{2}}{\lambda^{2}-9.89616^{2}}\;\left(\mathrm{for}\;\mathrm{fused}\;\mathrm{silica}\right)$$

The numerical values of RI for the fused silica for the operating wavelength range from 0.8–1.8 µm are tabulated in Table 1. 

## 3. Results and Discussion

**Figure 2:**
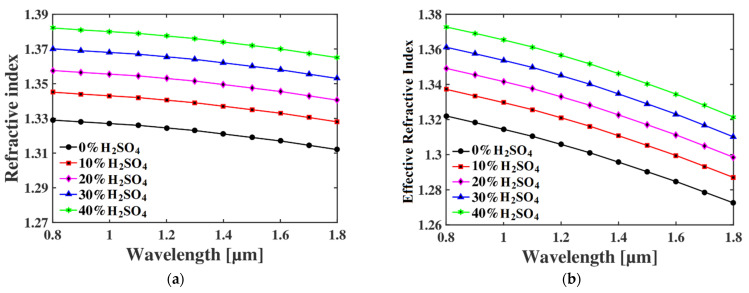
Graphical representation of the relationship between the (**a**) refractive index of different sulfuric acid of different concentrations with wavelength and (**b**) effective refractive index of the guided light with wavelength through the optical sensor.

The proposed PCF-based sulfuric acid detector was designed and analyzed using COMSOL Multiphysics Version 4.3, which is widely used by optoelectronics researchers. The whole process can be divided into two broad categories: designing the PCF and analyzing the guiding parameters’ values. In the designing phase, using the Cartesian coordinate system concept, the air holes of the core and cladding were designed. After completing the design of the PCF, the material properties of the base material, air, and the sensing analytes were provided in the simulation environment. The software used FEM to solve the differential equations, where the entire PCF structure was considered to consist of a finite number of triangular elements. In the analysis phase, Maxwell’s equations were applied to every element, and finally, the complete solution was generated. In order to ensure better accuracy, the wavelength-dependent refractive index of sulfuric acid was used, the values of which were taken from ref. [23]. The critical parameter for any optical sensor is its relative sensitivity, which indicates the slightest change in the sample detectable by that sensor. The higher the sensitivity, the better the performance of the sensor. The mathematical expressions for the relative sensitivity of the sensor and the total power that travels through the core of the fiber are expressed as follows [9,10,11]:

(3) $$r=\frac{n_{r}}{n_{eff}}\times P\%$$

(4) $$P=\frac{\int_{sample}R_{e}(E_{x}H_{y}-E_{y}H_{x})dxdy}{\int_{total}R_{e}(E_{x}H_{y}-E_{y}H_{x})dxdy}\times 100$$

where the relative sensitivity is represented by r, the effective refractive index (RI) of the guided signal and the sample are symbolized by *n_eff_* and *n_r_*, while *P* indicates the fraction of total electromagnetic (EM) power that travels through the core. In Equation (4), the electric and magnetic fields are represented by *E* and *H*, respectively. The subscript *x* and *y* represent the polarization modes when the signal travels in the z-direction. Figure 2 shows the wavelength-dependent RI of sulfuric acid for different concentration levels and the effective RI of the guiding light through the core of PCF. Figure 2a shows the wavelength-dependent RI of the sulfuric acid for concentrations from 0% to 40%. as reported in ref. [23]. It also indicates that the RI of the sulfuric acid decreases with the increase in wavelength. In addition, Figure 2b indicates the effective RI of the guiding light when the core of PCF is filled with the sample. The two figures are almost identical, as the combined refractive index (PCF material and sample) changes accordingly to the average RI of the core.

**Figure 3:**
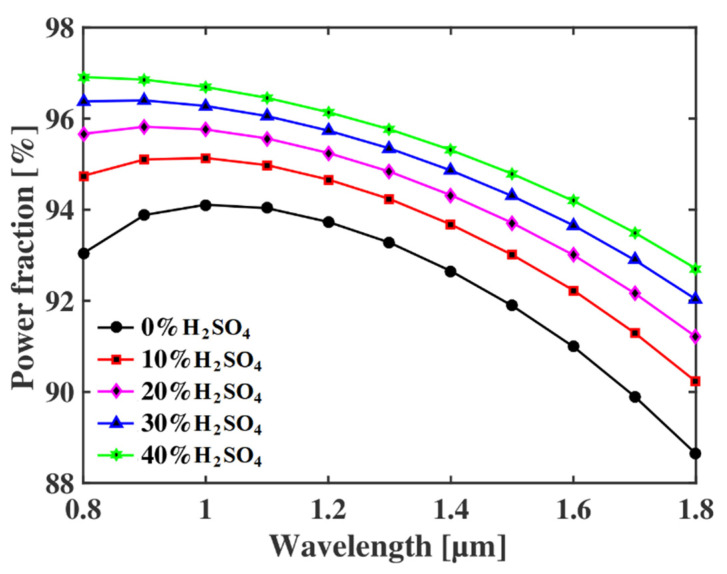
Power fraction of the proposed sulfuric acid detector at different operating wavelengths.

The power fraction of the proposed sensor at different wavelengths is shown in Figure 3 for different concentration levels of sulfuric acid at an optimum core diameter of Dc = 3.6 µm. The figure indicates that the power fraction increases for all H_2_SO_4_ concentrations till 1.1 µm, and after that, the power fraction decreases. As the relative sensitivity is proportional to P, the optimum wavelength is considered to be 1.1 µm. In addition, the value of P is maximum for 40% concentration of H_2_SO_4_ in water and lowest for pure water (RI = 1.33), as the refractive index is maximum for the acid and minimum for water.

**Figure 4:**
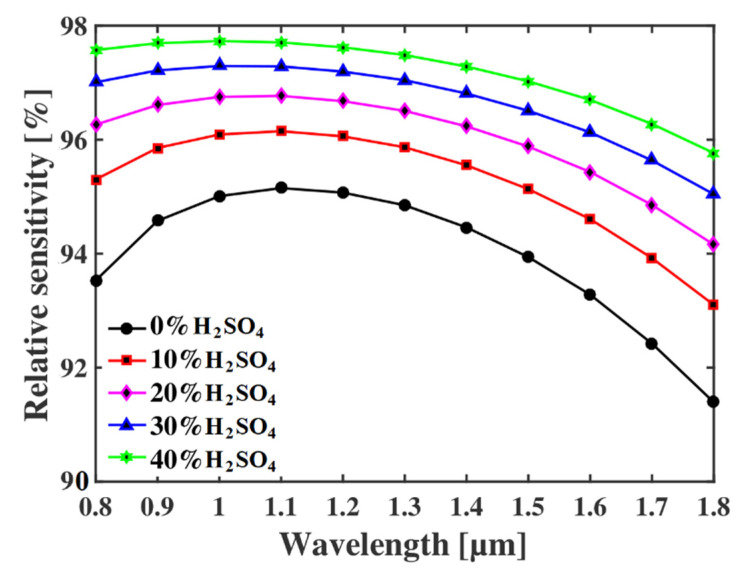
Relative sensitivity of the proposed detector for different wavelengths at *p* = 3 µm.

Finally, the relative sensitivity of that sensor is shown in Figure 4 for different concentrations of H_2_SO_4_ as a function of the operating wavelength. Since the value of ***r*** is directly proportional to P, the relative sensitivity characteristics are almost identical to Figure 3. For instance, the following Figure 4 shows that the relative sensitivities are 97.8%, 97.4%, 96.8%, 96%, and 95% for 40%, 30%, 20%, 10%, and 0% H_2_SO_4_ at a wavelength of 1.1 µm, which are better than the previously proposed sensor in Ref. [23], where the maximum reported sensitivity was only around 80%. 

**Figure 5:**
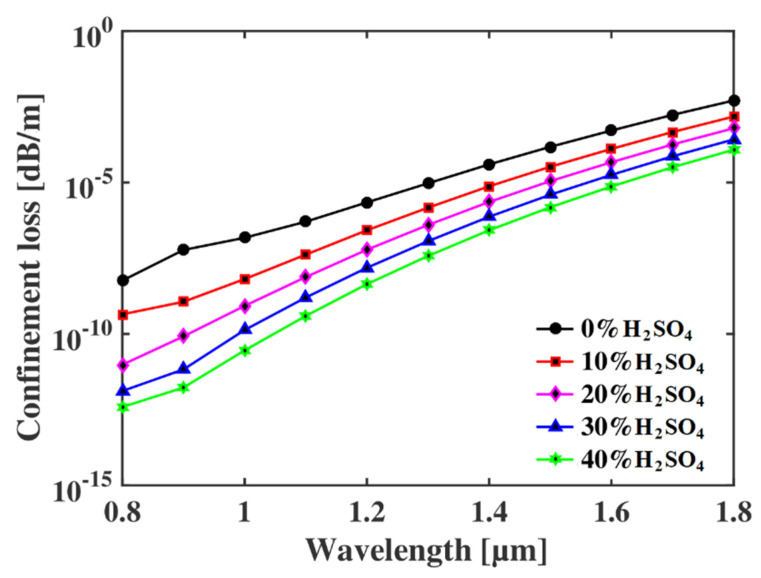
Confinement loss of the proposed sensor at different wavelengths for different concentrations of H_2_SO_4_.

Next, we investigated the loss profile of the proposed sensor under various operating conditions. When the EM wave travels through the core and interacts with the sample inside the core, a fraction of light trapped by the air holes surrounds the core. This type of loss is called the confinement loss and it occurs in every type of optical waveguide and is calculated by using the following expression [14,15,18]:

(5) Lc=40πln(10)λIm(neff)×106dB/m

where *λ* stands for the operating wavelength and Im(*n_eff_*) is the imaginary part of the effective refractive index. The relationship between the confinement loss and the propagating EM waves is shown in Figure 5. It is evident that the loss is higher for the lower refractive indexed sample. This occurs as the tendency of the EM wave is to propagate through the higher refractive indexed region. As the RI of the sample decreases with the increase in water mixture, less light travels through the core, and the confinement loss increases. This phenomenon is already observed in Figure 4, according to which the core power decreases at higher operating wavelengths, meaning that more light diverges from the core and is trapped by the cladding air holes. As a result, the confinement loss is higher for higher operating wavelengths. At optimum conditions, the proposed sensor exhibits low confinement loss of around 10^−7^ to 10^−9^ for different concentrations of analyte at 1.1 µm.

**Figure 6:**
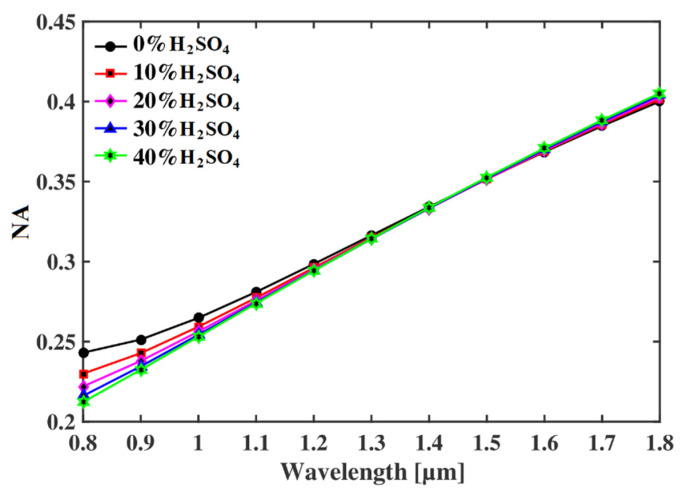
NA of the proposed sensor as a function of operating wavelength.

Another key parameter to evaluate the proposed sensor is the numerical aperture (*NA*), which is a dimensionless parameter and varies from 0.1 to 0.5. The *NA* is the maximum incident light angle from the light source that is accepted by the optical fiber. The relationship to calculate the *NA* of the proposed sensor is as follows [14]:

(6) $$NA=\frac{1}{\sqrt{1+\frac{\pi A_{eff}f^{2}}{c^{2}}}}\approx \frac{1}{\sqrt{1+\frac{\pi A_{eff}}{\lambda^{2}}}}$$

where *λ* is for the wavelength of the EM signal and *A_eff_* is the effective area of the guided light. The relationship between the *NA* of the proposed sensor and the wavelength is reported in Figure 6, which indicates that the *NA* increases with the increase in *λ*. The dominating factor in Equation (4) is the operating wavelength and due to this, the *NA* is higher at a longer wavelength. Though the effective area increases at a larger wavelength, the *NA* is mainly dependent on *λ*. At optimum wavelength, the proposed optical sensor offers a high numerical aperture of 0.28, which, to the best of our knowledge, is not previously reported in the literature.

**Figure 7:**
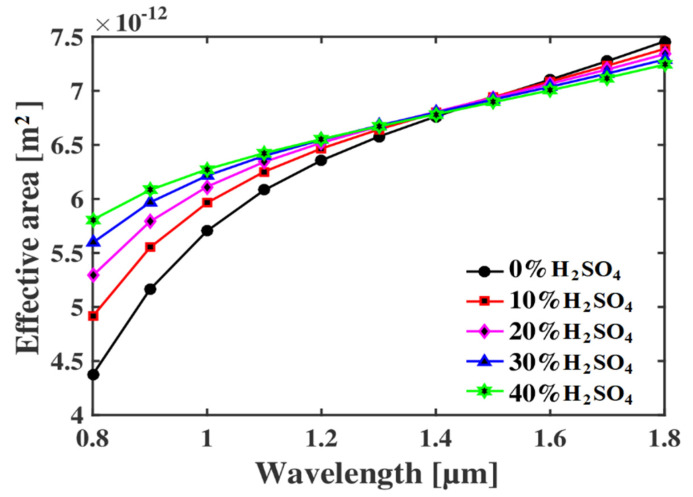
Relationship between the effective area and the operating wavelength at p = 3 µm.

In the PCF-based sensor, the effective area is defined as the total area of the sensor where the signal actually propagates from source to destination. The effective area can be calculated by using the given equation [14] as follows:

(7) $$\;A_{eff}=\frac{{\left[\int I\left(r\right)rdr\right]}^{2}}{{\left[\int I^{2}\left(r\right)dr\right]}^{2}}$$

where *A_eff_* is the effective area, and $I\left(r\right)={\left|E_{t}\right|}^{2}$, is the electric field intensity of the optical sensor. The effective area versus operating wavelengths is shown in Figure 7. It is quite evident that the effective area increases as the divergence of EM wave from the core increase for the larger wavelength. For sensing applications, the smaller effective area is desirable so that the maximum light signal can interact with the analyte and the relative sensitivity increases. Here, the proposed sensor offers a very small effective area in the range of 6 × 10^−12^–6.4 × 10^−12^ m^2^ at an operating wavelength of 1.1 µm for different concentrations of H_2_SO_4_.

**Figure 8:**
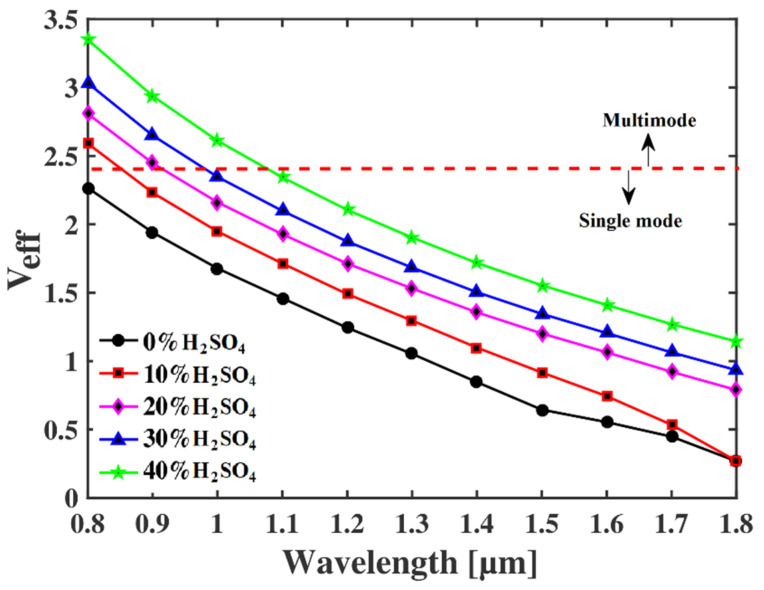
*V_eff_* of the proposed sensor for different operating wavelengths.

The single-mode propagation parameter, which is also termed as V parameter or *V_eff_* is also calculated. This parameter of any PCF informs whether the fiber will experience multimodal distortion or not when guiding an EM signal of a particular wavelength/frequency [24,25]. The fundamental equation of extracting *V_eff_* of an optical fiber is as follows [24]:

(8) $$V_{eff}=\frac{2\pi }{\lambda }R\sqrt{n_{co}^{2}-n_{cl}^{2}}$$

where *R* is the core radius, and the refractive index of core and cladding are indicated by *n_co_* and *n_cl_*, respectively. The only condition to be a single-mode fiber is that the numerical value of this parameter must be less than or equal to 2.405. If the value exceeds the threshold value, then the fiber will experience modal distortion. The visual representation of *V_eff_* of the proposed H_2_SO_4_ concentration detector is shown in Figure 8 for different operating wavelengths. It is clear that the value of *V_eff_* decreases with the increase in *λ*. As the refractive index difference between the core and cladding reduces with the increase in operating wavelength, the value of *V_eff_* also reduces gradually. However, the value of this parameter is less than 2.405 at *λ* = 1.1 µm for all different concentrations of sulfuric acid samples. Therefore, the proposed sensor guarantees multimodal distortion and less light reception at the receiving end of the sensing system.

**Figure 9:**
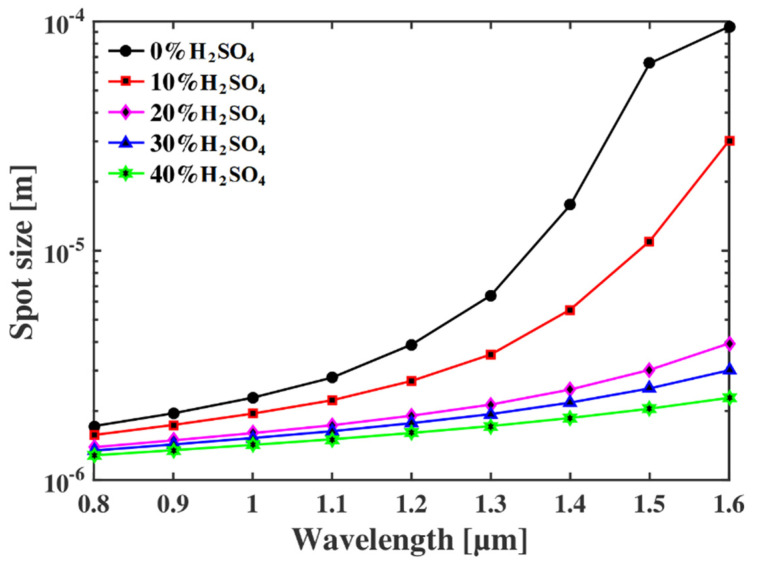
The spot size of the proposed sensor for different operating wavelengths.

Now the spot size of the proposed optical fiber-based sensor is investigated for different operating conditions. A larger spot size is a desirable parameter for sensing applications, as it indicates better light–analyte interaction in the sensor itself. The mathematical relation to evaluating the spot size is as follows [26,27,28]:

(9) $$W_{eff}=R\times \left(0.65+1.619\times V^{-1.5}+2.879\times V^{6}\right)$$

where *W_eff_* is the effective spot size, *R* is the radius of the core, and *V* is the normalized V-parameter. The relationship between the spot size and the operating wavelength is shown in Figure 9, according to which the values of this parameter increase with the operating wavelength. As all the parameters of the proposed sensor are reported at an operating wavelength of 1.1 µm, we found that the value of spot size at this operating condition is 1.5 µm, which is better than other reported sensors.

Table 2 shows a detailed comparison based on important results between the proposed study and past studies. Indeed, the prime characteristic of any sensor is its relative sensitivity, and the following table confirms that the proposed sensor is far better than the previously reported studies. Moreover, the other parameters are also comparable with the recent research findings.

Finally, the potential fabrication techniques of the proposed sensor are elucidated here. The proposed sensor is very simple to realize as all the air holes are circular in shape. It has been reported that circular-type air holes can be produced with high accuracy in the laboratory setting [29,30]. There are several methods to fabricate the PCFs—namely, the stack and draw technique, sol–gel method, drilling, and stacking, extrusion, etc. It is well known that in order to fabricate these types of sensors, the extrusion technique is the most suitable due to its higher accuracy and reliability [31].

## 4. Conclusions

In conclusion, we proposed a highly sensitive PCF-based optical sensor to accurately determine the concentration level of sulfuric acid in water. This sensor was designed by using all circular structures in the core, as well as the cladding area, to reduce fabrication complexity. The simulation results were based on FEM, which provides favorable sensing and guiding properties to be extracted from the proposed sensor. At optimum structural and operating conditions, the proposed PCF-based sensor offers a very high relative sensitivity of more than 97%, low confinement loss of 10^−9^ dB/m, a high numerical aperture of 0.28, and finally, optimal spot size. We strongly believe that the proposed sensor design will open new avenues for researchers working in the field of optical sensing.

## Figures and Tables

**Table 1:** Refractive index of fused silica for different operating wavelengths.

**Wavelength** **(µm)**	0.8	0.9	1.0	1.1	1.2	1.3	1.4	1.5	1.6	1.7	1.8
**RI of fused silica**	1.453	1.452	1.45	1.449	1.448	1.447	1.446	1.445	1.443	1.442	1.44

**Table 2:** Comparison between the guiding and sensing properties of some of the recently proposed schemes.

Ref	Year	Sensing Analyte	Frequency/Wavelength	Sensitivity (%)	Confinement Loss (dB/m)	Numerical Aperture	Single-Mode Propagation
		Methanol	1.55 µm	75.22	3.29 × 10^−10^	0.32	Yes
		Ethanol		82.52	1.50 × 10^−10^	0.3	Yes
[14]	2021	Propanol		86.74	1.35 × 10^−10^	0.29	Yes
		Butanol		88.34	9.33 × 10^−10^	0.28	No
		Water	1.33 µm	50	4.25 × 10^−10^	---	N/A
[17]	2016	Ethanol		55.83	8.72 × 10^−10^	---	N/A
		Benzene		59.07	2.56 × 10^−10^	---	N/A
		0% H_2_SO_4_	1.5 µm	60.7	8.48 × 10^−19^	---	N/A
		10% H_2_SO_4_		61.4	3.48 × 10^−19^	---	N/A
		20% H_2_SO_4_		62.0	1.96 × 10^−19^	---	N/A
[23]	2019	30% H_2_SO_4_		62.70	3.25 × 10^−20^	---	N/A
		40% H_2_SO_4_		63.4	1.42 × 10^−20^	---	N/A
		0% Petrol	2.8 THz	89.40			N/A
		20% Petrol		88.85			N/A
		40% Petrol		88.15			N/A
[24]	2020	60% Petrol		87.50	Order of 10^−8^	0.36	N/A
		80% Petrol		86.90			N/A
This work		0% H_2_SO_4_	1.1 µm	95.0	1.39 × 10^−7^		Yes
		10% H_2_SO_4_		96.0	5.87 × 10^−8^		Yes
		20% H_2_SO_4_		96.8	1.65 × 10^−8^		Yes
	2022	30% H_2_SO_4_		97.4	7.33 × 10^−9^	0.28	Yes
		40% H_2_SO_4_		97.8	2.53 × 10^−9^		Yes

## Data Availability

The data that support the findings of this study are available upon request from the corresponding author. The data are not publicly available due to privacy or ethical restrictions.
